# The Role of Melanin Concentrating Hormone (MCH) in the Central Chemoreflex: A Knockdown Study by siRNA in the Lateral Hypothalamus in Rats

**DOI:** 10.1371/journal.pone.0103585

**Published:** 2014-08-01

**Authors:** Ningjing Li, Eugene Nattie, Aihua Li

**Affiliations:** Department of Physiology and Neurobiology, Geisel School of Medicine at Dartmouth, Lebanon, New Hampshire, United States of America; Hosptial Infantil Universitario Niño Jesús, CIBEROBN, Spain

## Abstract

Melanin concentrating hormone (MCH), a neuropeptide produced mainly in neurons localized to the lateral hypothalamic area (LHA), has been implicated in the regulation of food intake, energy balance, sleep state, and the cardiovascular system. Hypothalamic MCH neurons also have multisynaptic connections with diaphragmatic motoneurons and project to many central chemoreceptor sites. However, there are few studies of MCH involvement in central respiratory control. To test the hypothesis that MCH plays a role in the central chemoreflex, we induced a down regulation of MCH in the central nervous system by knocking down the MCH precursor (pMCH) mRNA in the LHA using a pool of small interfering RNA (siRNA), and measured the resultant changes in breathing, metabolic rate, body weight, and blood glucose levels in conscious rats. The injections of pMCH-siRNA into the LHA successfully produced a ∼62% reduction of pMCH mRNA expression in the LHA and a ∼43% decrease of MCH levels in the cerebrospinal fluid relative to scrambled-siRNA treatment (P = 0.006 and P = 0.02 respectively). Compared to the pretreatment baseline and the scrambled-siRNA treated control rats, knockdown of MCH resulted in: 1) an enhanced hypercapnic chemoreflex (∼42 & 47% respectively; P < 0.05) only in wakefulness; 2) a decrease in body weight and basal glucose levels; and 3) an unchanged metabolic rate. Our results indicate that MCH participates not only in the regulation of glucose and sleep-wake homeostasis but also the vigilance-state dependent regulation of the central hypercapnic chemoreflex and respiratory control.

## Introduction

Melanin concentrating hormone (MCH), a 17-amino acid neuropeptide, was originally isolated from the pituitary gland of salmon where it controls skin pigmentation [1]. In mammals, MCH is mainly synthesized in neurons of the lateral hypothalamus and zona incerta in the central nervous system (CNS), where it is posttranslationally cleaved from a larger precursor molecule, prepro-MCH (pMCH). The axons of hypothalamic MCH-containing neurons project diffusely to multiple sites in the CNS and the MCH receptors are widely distributed throughout the brain [2], [3], suggesting multiple functions for MCH.

Neuropharmacological studies in rats and an MCH-overexpressing mouse model have demonstrated important roles of MCH in the regulation of body weight and food intake [4], [5]. MCH also participate in the balance of the sleep-wake cycle [6], [7]. Recent studies showed that central administration of MCH into lateral ventricle (i.c.v.) or nucleus tractus solitarius (NTS) caused depressor and bradycardiac responses in both anesthetized and conscious rats [8], [9]. Intrathecal injection of MCH produced a dose-dependent hypotension, bradycardia, and sympathetic depression, and attenuated the sympathetic response to stimulation of peripheral (anoxia, ∼4.4% O_2_) or central (hyperoxic hypercapnia 10% CO_2_/93% O_2_) chemoreceptors [10]. This evidence suggested a role of MCH in autonomic functions.

Anatomical studies have suggested a role for MCH in respiratory control. Injection of a transneuronal tracer into the diaphragm showed that phrenic motoneurons are innervated by both hypothalamic MCH and orexin neurons [11]. MCH neurons send projections to many brain sites that contain putative central chemoreceptors, such as the locus coeruleus (LC), the dorsal raphe, the NTS, and the area of retrotrapezoid nucleus (RTN) [3], [12]–[15]. In addition, in the lateral hypothalamic area (LHA), MCH neurons are closely intermingled with chemosensitive orexin neurons, and these two types of neurons are interconnected [11], [16]–[19]. Orexin induces depolarization of MCH neurons by evoking a direct inward current and by increasing excitatory synaptic activity [20], [21]. MCH reduces glutamate release at presynaptic terminals and blocks orexin-A mediated enhancement of action potential generation in orexin neurons [22]. Orexin is known to participate in the regulation of cardiorespiratory function and in the central hypercapnic chemoreflex [23], [24] while the role of MCH in these functions is poorly understood. Here we hypothesize that MCH also participates in regulation of the central chemoreflex and that suppression of MCH activity will significantly increase the central hypercapnic chemoreflex.

RNA interference (RNAi) is a mechanism for highly selective and reversible gene suppression [25], and the small interfering RNA (siRNA) can bind to the specific messenger RNA (mRNA) molecules and decrease their activity via preventing an mRNA from producing a protein [25]. In rat using siRNA, Chen *at al.* successfully knocked down the prepro-orexin in the LHA, the same area where the MCH neurons reside [26]. In this study, we utilized a pool of siRNA to knock down the expression of the precursor gene, pMCH, which encodes the production of MCH in the LHA, to assess the role of MCH in the central chemoreflex. We measured the hypercapnic ventilatory response, together with body weight, body temperature, metabolic rate, glucose levels in wakefulness and sleep before and after knockdown and in controls injected with scrambled siRNA.

## Methods

### Ethical Approval

All animals were treated humanely and all experiments were conducted in strict accordance with the guidelines of the National Institutes of Health for animal use and care using a protocol approved by the Institutional Animal Care and Use Committee (IACUC) at the Geisel School of Medicine at Dartmouth College. All surgical procedures were performed under ketamine/xylazine anesthesia, and all efforts were made to minimize suffering.

### Animals

A total of 37 adult Sprague–Dawley male rats (250–300 g; Harlan Laboratories, IN) were used in this study. All the animals were individually housed under 12 h: 12 h light: dark cycle (lights on at 12∶00 AM, off at 12∶00 PM) at 22±1°C. Food and water were provided ad libitum. At the conclusion of the experiments, the rats were killed by intraperitoneal (i.p.) injection of a euthanasia solution, Euthasal (0.3 ml/kg, Virbac AH, Inc, TX, USA).

### Experimental design

Two sets of experiments were designed to evaluate the effects of pMCH knockdown via pMCH-siRNA, and they are: 1) to verify, *in* *vitro*, the effectiveness of the pMCH siRNA treatment and examine the levels of MCH peptide in the CSF following the pMCH siRNA administration, and 2) to evaluate, *in* *vivo*, the effects of a lower level of MCH in the CNS on body weight, glucose homeostasis, metabolic rate, and hypercapnic ventilatory response in wakefulness and sleep.

### Experiment 1

In the first set of experiments, we verified the target effects of the pMCH siRNA by performing a real-time PCR for mRNA expression of pMCH, orexin-A, and orexin receptor 1 (OX1R) in the LHA, and a fluorescent enzyme immunoassay (EIA) for the protein levels of MCH in the CSF.

#### Surgery and siRNA injections

The animals were randomly divided into 3 groups with 5 animals in each group. After intramuscular (i.m.) administration of ketamine (100 mg/kg, Putney, Inc. Portland, ME, USA) and xylazine (15 mg/kg, Lloyd Labs, Walnut, CA, USA) cocktail, rats in Group 1 were bilaterally injected with a pool of pMCH siRNA (0.06 nmol in 0.3 µl water; ID nos: s128229, s217872, s217873, Ambion, Applied Biosystem, USA; Table 1). Animals in Group 2 received injections of scrambled sequences (scrambled siRNA), which have no homology to known rat genes, as control (0.06 nmol in 0.3 µl water; Ambion In Vivo Negative Control #1 siRNA, Applied Biosystem, USA; Table 1). Rats in Group 3 received injections with artificial CSF (aCSF, 0.3 µl). All injections were made into the LHA using a Hamilton syringe with two injections on each side. Coordinates [27] for the LHA target site were: 3–3.4 mm caudal from bregma and 1–2 mm lateral to the midline, with a depth of 8–8.8 mm from the surface of the skull. Forty-eight hours after treatment, the animals were sacrificed with an overdose of Euthasal (0.3 ml/kg, i.p.). 48 hours post injection, the hypothalamus was quickly dissected out, weighed, and immediately immersed in RNAlater RNA stabilization reagent (5 ml, Qiagen, Valencia, CA, USA) and subsequently stored at 4°C until processed for RNA extraction.

**Table 1:** Sequences of siRNA.

siRNA ID	Sense	Antisense
128229	CGUAGAAGACGACAUAGUAUU	UACUAUGUCGUCUUCUACGUU
217872	AGCAGAAUCUCGUAACUCAUU	UGAGUUACGAGAUUCUGCUUG
217873	GGAGAGAUUUUGACAUGCUUU	AGCAUGUCAAAAUCUCUCCUU
*In Vivo*NegativeControl #1	UAACGACGCGACGACGUAA	UUACGUCGUCGCGCGUCGUUA

#### RNA extraction and Real-time PCR

Total RNA was isolated from the dissected hypothalamic tissue using QIAzol lysis reagent according to the manufacturer's manual (Invitrogen, Carlsbad, CA, USA) and purified on RNeasy Lipid Tissue Mini Kit columns (Qiagen, Valencia, CA, USA) with on-column DNase digestion using the RNase-Free DNase set (Qiagen, Valencia, CA, USA). Six hundred ng of total RNA was reverse-transcribed using the iScriptc DNA synthesis kit (Bio-Rad, Hercules, CA, USA) according to the manufacturer's directions. Relative mRNA expression levels of genes of interest were measured using the 5′-fluorogenic nuclease assay in real-time quantitative PCR using TaqMan chemistry on the ABI 7300 Prism real-time PCR instrument (Applied Biosystems, Carlsbad, CA, USA). The pMCH, orexin-A, OX1R, and cyclophilin A (cyc A) primer/MGB probe sets were obtained from Applied Biosystems assays-on-demand (vendor ID nos: Rn00561766, Rn00565995, Rn00565032 and Rn00690933, respectively). PCR was conducted using the following cycle parameters: 50°C for 2 min, 95° for 12 min for the first cycle, followed by 40 cycles of 95° for 20 seconds and 60° for 1 min. Analysis was performed using the sequence detection software supplied with the ABI 7300. The relative quantification was performed using the comparative threshold cycle (Ct) with the ΔCt values determined by subtracting the values of the reference control gene from the target gene Ct values. The difference in the mRNA expression levels between the groups was expressed as 2−ΔΔCt, where ΔΔCt equals the difference in ΔCt between the treatment and scrambled siRNA rats. The levels of the gene expression were calculated and presented as percentage (%) of the fold change.

#### CSF collection and EIA

CSF was collected from the cistern magna via puncture of the atlanto-occipital membrane with a 28½ G insulin syringe (1 mL, BD SafetyGlide, USA) under anesthesia (ketamine/xylazine cocktail, 100/15 mg/kg, i.p.) 72 hrs post-injection in all three groups and was immediately frozen and stored in −80°C. For better detection, CSF was 1∶1 diluted in the 1x assay buffer provided in the commercial Fluorescent EIA Kit (FEK-070-47, Phoenix Pharmaceuticals, CA, USA). The concentration of MCH was measured according to the manufacturer’s manual of the kit. Standard curves of MCH were prepared for each assay by mixing different concentrations of non-biotinylated MCH with a fixed concentration of biotinylated-MCH peptide. The absorbance was measured in a 96-well plate reader (BioTek Synergy 2, Winooski, VT, USA). Absorbance results (pg/ml) were log transformed.

### Experiment 2

#### Surgery and siRNA Injections

Twenty two rats were anesthetized using the method described above for Experiment 1 and surgically implanted with electroencephalogram (EEG) and electromyogram (EMG) electrodes (PlasticsOne Inc., VA, USA) and a telemetric temperature probe (Data Sciences International, MN, U.S.A) placed in the abdominal cavity. The detailed surgical procedures have been described in an earlier paper [28]. Briefly, three EEG electrodes were screwed onto the skull and two EMG electrodes were inserted under the dorsal neck muscles with all electrode wires connected to a six-prong plastic pedestal (Plastics One Inc., VA, USA), which was attached to the skull by cranioplastic cement (Lang Dental Manufacture, Co., Inc., Wheeling, IL, USA). Two holes were drilled on each side of the skull with coordinates: 3–3.4 mm caudal from bregma and 1–2 mm lateral to the midline to allow access to the LHA later [27]. A sterile telemetry temperature probe (TA-F20, Data Sciences, St Paul, MN, USA) was implanted into the peritoneal cavity. After the incisions were sutured, the animals were allowed to recover for at least 7 days.

After the pretreatment baseline experiments, a pool of siRNA (pMCH siRNA or scrambled siRNA, 0.06 nmol in 0.3 µl water mixed with red IX retrobeads (LumaFluor, Inc. Durham, NC, USA) was injected into the LHA in rats under general anesthesia. Coordinates for the LHA target site were: 3–3.4 mm caudal to bregma; 1–2 mm lateral to the midline and 8–8.8 mm from the surface of the skull [27].

#### Blood glucose and Body Weight

Body weight and blood glucose level were measured before each physiological experiment. To collect blood sample for glucose measurement, a small cut was made near the end of the rat tail, and the drops of blood were collected directly on the test strip placed in the blood glucometer (Contour, Bayer HealthCare LLC, USA).

#### Ventilation, Oxygen Consumption and Temperature Measurement

The methods used to measure ventilation (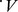
_E_), body temperature (T_b_) and oxygen consumption (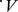
_O2_) in conscious rats were those commonly used in our lab [29]. Briefly, we used the whole body plethysmograph with a volume of 7.6 L and a 3.5 L top designed to protect the head pedestal. The chamber was connected to a similarly sized reference chamber by a high resistance port. Analog output from the pressure transducer was sampled at 150 Hz and converted into digital signals by using the DATAPAC 2K2 system (RUN Technologies, Mission Viejo, CA, USA) on a computer. The rate of gas inflow was maintained at ≥1.4 L min^−1^ with the plethysmograph at atmospheric pressure. CO_2_ and O_2_ fractions were sampled from the outflow at ∼100 ml min^−1^ by a CO_2_ and O_2_ gas analyzer (AEI Technologies, INC., Pittsburgh, PA, USA). The plethysmograph was calibrated by obtaining pressure response data from five 0.3 ml air injections using a 1 ml syringe before each experiment began. Breath-by-breath analysis was performed using the pressure deflections and the respiratory cycle time for each breath.

Tidal volume (V_T_) and respiratory frequency (f_R_) were calculated per breath to estimate ventilation per breath. V_T_ was calculated by using the equation published in 1970 [30]. By using EEG and EMG criteria, 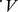
_E_ was calculated for each animal during both quiet wakefulness and NREM sleep and was expressed as mean values. 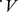
_O2_ was calculated by application of the Fick principle using the difference in O_2_ content between inflow and outflow air and the flow rate as described previously [29]. Core body temperature was measured continuously using the signals from the telemetric temperature probe surgically placed in the abdomen. The chamber temperature was measured using a thermometer inside the plethysmograph.

### Determination of vigilance state

Raw EEG and EMG signals from electrodes on the skull and in the neck muscles were sampled at 150 Hz, filtered at 0.3–70 and 0.1–100 Hz, respectively, and were recorded by the DATAPAC system across the experimental period. A fast Fourier transform was performed on the EEG and EMG signals at 4.0 s long epochs to determine the vigilance states as previously described [28], [31]. The vigilance states were categorized as wakefulness, NREM, and REM sleep. Since not all rats have data in REM sleep in both air and 5% CO_2_ breathing conditions the data in REM sleep are omitted here.

### Experimental protocol

The physiological measurements were performed before (baseline) and 72 hour after being injected with siRNA (scrambled siRNA or pMCH siRNA) during the early dark period. For the ventilatory hypercapnic chemoreflex (CO_2_ response), the rats were allowed to acclimate for 1 h under room air (23–25°C) in the plethysmograph chamber. In order to obtain data in both wakefulness and NREM sleep we recorded baseline data in room air for 45 min, and then 5% CO_2_ for another 45 min. After pretreatment baseline experiments, the rats were injected with siRNA (scrambled siRNA or pMCH siRNA) through the two pre-drilled holes on the skull during the 1st surgery under general anesthesia.

### Anatomy analysis

Upon completion of the experiments, the rats were euthanized with an overdose of Euthasal. The whole brain was dissected, frozen, and sectioned at 30 µm thickness with a Reichert–Jung cryostat (Leica Microsystems, Buffalo Grove, IL, USA). The sections were then examined under fluorescence microscopy (Olympus, NJ, USA) to verify the injection sites. The third ventricle and the fornix were used as the landmarks to determine injection sites in the LHA.

### Statistical analysis

All data are presented as mean ± standard error of the mean (S.E.M.). We used SigmaPlot 11.0 (Systat Software Inc., San Jose, CA, USA) to apply one-way ANOVA separately to assess the effects of treatments on mRNA expression of pMCH, orexin-A, and OX1R with application of Bonferroni's test for post hoc comparisons. For EIA data, we applied unpaired t-test to MCH levels in the CSF after pMCH siRNA or scrambled siRNA injections. For analysis of 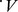
_E_/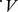
_O2_ and Δ
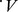
_E_, we applied two way RM ANOVA with gas and treatment as the two factors then applying Bonferroni's post hoc test to compare pre- and post- treatment; for comparisons between treatments, we used a two way ANOVA with treatment and gas as factors then applying Bonferroni’s test for post hoc comparisons. To further investigate whether treatment effects were dependent on gas components or vigilance state, we applied a three way ANOVA with treatment, gas and vigilance state as factors. For the analysis of body weight, glucose levels and sleep we applied paired t-test to assess the effects before and after siRNA injections and one way ANOVA with Bonferroni's post hoc test to compare effects among groups.

## Results

### Anatomic Analysis

**Figure 1:**
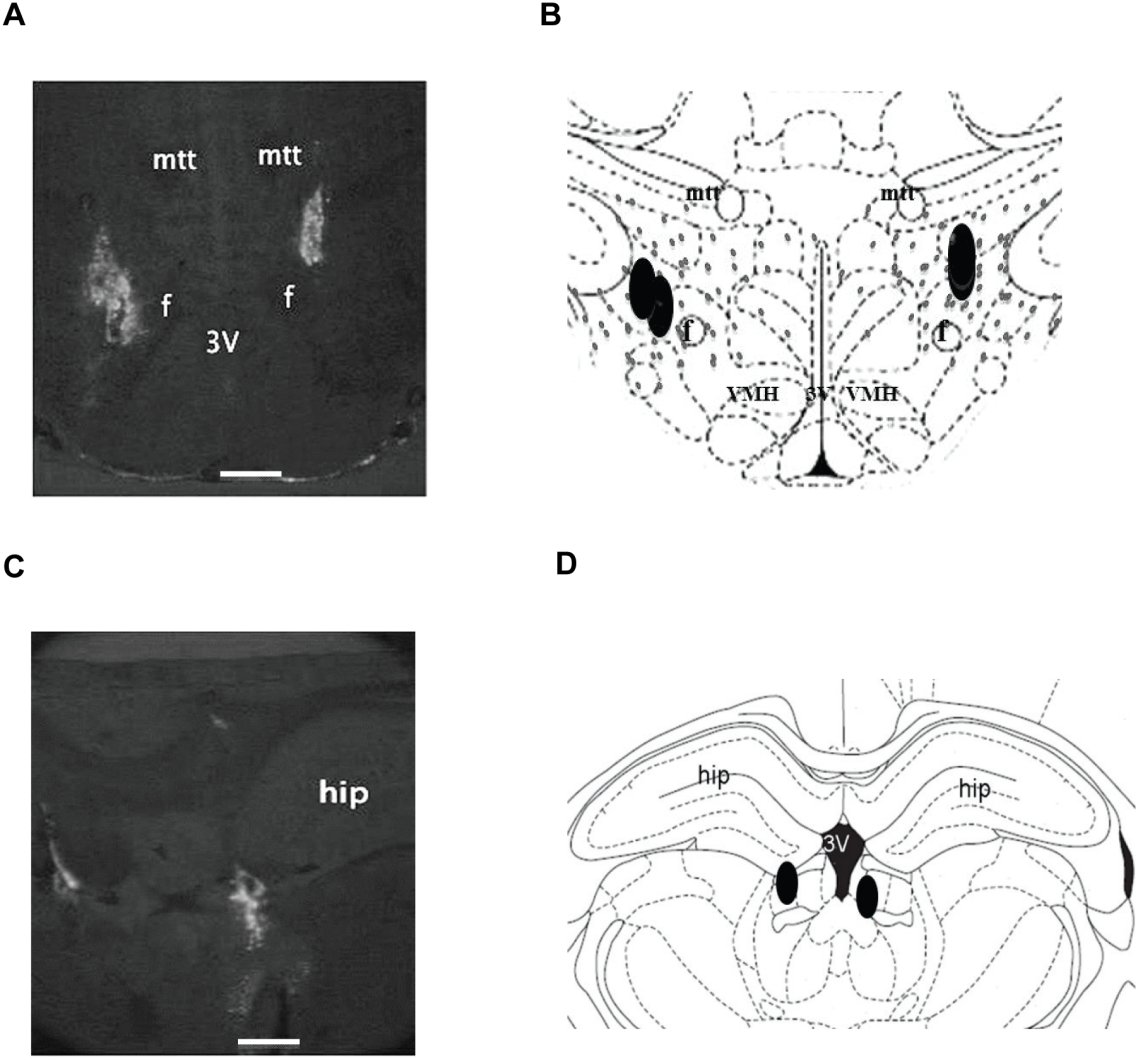
Anatomic locations of the injection sites. (A) Representative cross section showing the injection sites (fluorescent beads) located within the lateral hypothalamic area (LHA), two injections on each side (note: two injections were overlapping on one side). (B) Schematic corresponding section of A illustrating the injection sites (black) and the distribution of MCH neurons (small grey dots, modified from (Paterson & Hahn 2010)) in the LHA. (C) Representative section and (D) the schematic corresponding section from a rat showing the injection sites localized outside the LHA in the hippocampus. Abbreviations: 3v, third ventricle; f, fornix; hip, hippocampus; mtt, mammillothalamic trac; VMH, ventromedial hypothalamus. Scale bar represents 0.5 mm.

A representative example of the anatomic locations of siRNA injection sites (bilateral) indicated by fluorescent beads (bright white) in the LHA is shown in Fig. 1A. There is noticeable overlap among injection sites. The schematic coronal cross-section of the hypothalamus demonstrates the relationship of the injection sites (black filled symbols) and the distribution of hypothalamic MCH neurons (small grey dots) drawn according to the MCH-immunoreactive perikarya staining in the LHA of adult rats [16], [27] (Fig. 1B). Injection sites found outside the LHA, e.g., Fig. 1C & D, were considered as off-target injections.

### mRNA expression

**Figure 2:**
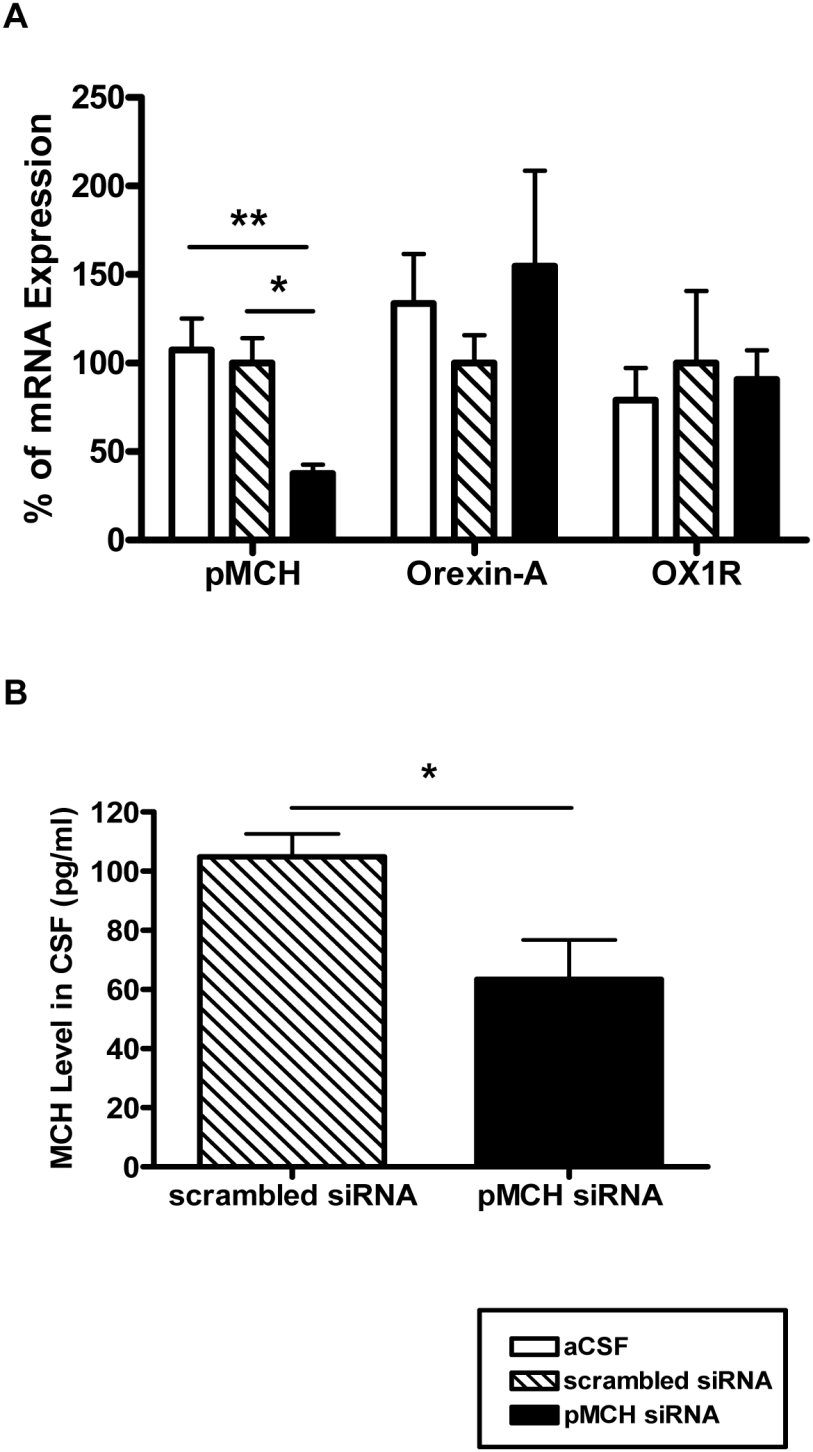
The effects of knockdown of pMCH mRNA expression on the expression levels of pMCH, orexin-A and orexin receptor 1 (OX1R) mRNA in the LHA (A) and MCH peptide levels in cerebrospinal fluid (CSF; B). (Mean ± S.E.M.). The levels of pMCH, orexin-A, and OX1R mRNA expression in the LHA were measured by RT-PCR 48 hrs post injections of aCSF (open bars), pMCH siRNA (filled bars) or scrambled siRNA (hatched bars) into the LHA (A; N = 5 each group; one way ANOVA, Bonferroni’s post hoc test). MCH peptide levels in the CSF were measured by EIA 72 hrs post injections of pMCH siRNA (black bar) or scrambled siRNA (hatched bar) (N = 5 & 6 respectively; unpaired t-test). Significant difference is indicated with *(P<0.05) or **(P<0.01).

Real-time PCR data showed an average 62% down-regulation of pMCH mRNA 48 hours post injection of pMCH siRNA into the LHA relative to that of both scrambled siRNA and aCSF control groups (Fig. 2A; P = 0.006, one way ANOVA, N = 5 each group). Bonferroni’s post hoc test showed pMCH mRNA was significantly lower in the pMCH siRNA group compared to that in the scrambled siRNA group (N = 5, P<0.05) or to that in the aCSF group (N = 5, P<0.01). There was no significant difference in pMCH mRNA expression between the scrambled siRNA group and the aCSF group (N = 5, P>0.05). pMCH mRNA levels in the pMCH siRNA group were: mean, 37.5% of the controls; range, 26.3%–48.7%; controls being the scrambled siRNA group: mean, 100%; range, 69.7%–131.7%; in the aCSF group: mean, 107.3% of the controls; range, 66.85%–146.15%. We also tested whether knocking down pMCH has non-specific effects on the adjacent neuronal groups, e.g., orexin neurons, since MCH and orexin neurons are intermingled within the LHA (Van den Pol, et al 2004; Rao et al, 2008). There was a trend towards an increase (∼54%) in orexin-A mRNA levels in the pMCH siRNA treated group; it was not statistically significant. No significant changes were observed in OX1R mRNA levels in any group. These data demonstrated that pMCH siRNA has specific targeted effects of on pMCH mRNA.

### MCH level in CSF

Fluorescent EIA showed that knocking down pMCH with pMCH siRNA also resulted in a ∼43% decrease of MCH levels in CSF compared to that of scrambled siRNA treated rats (mean ± s.e.m.; Fig. 2B, N = 5, 6, respectively; P = 0.02, unpaired t-test). MCH peptide levels (1∶1 diluted in 1x assay buffer) were 104.9±7.8 pg/ml and 60.0±9.7 pg/ml in the CSF in scrambled siRNA group and pMCH siRNA group, respectively, and the lowest level was observed at 48 hr post treatment.

### Hypercapnic chemoreflex

**Figure 3:**
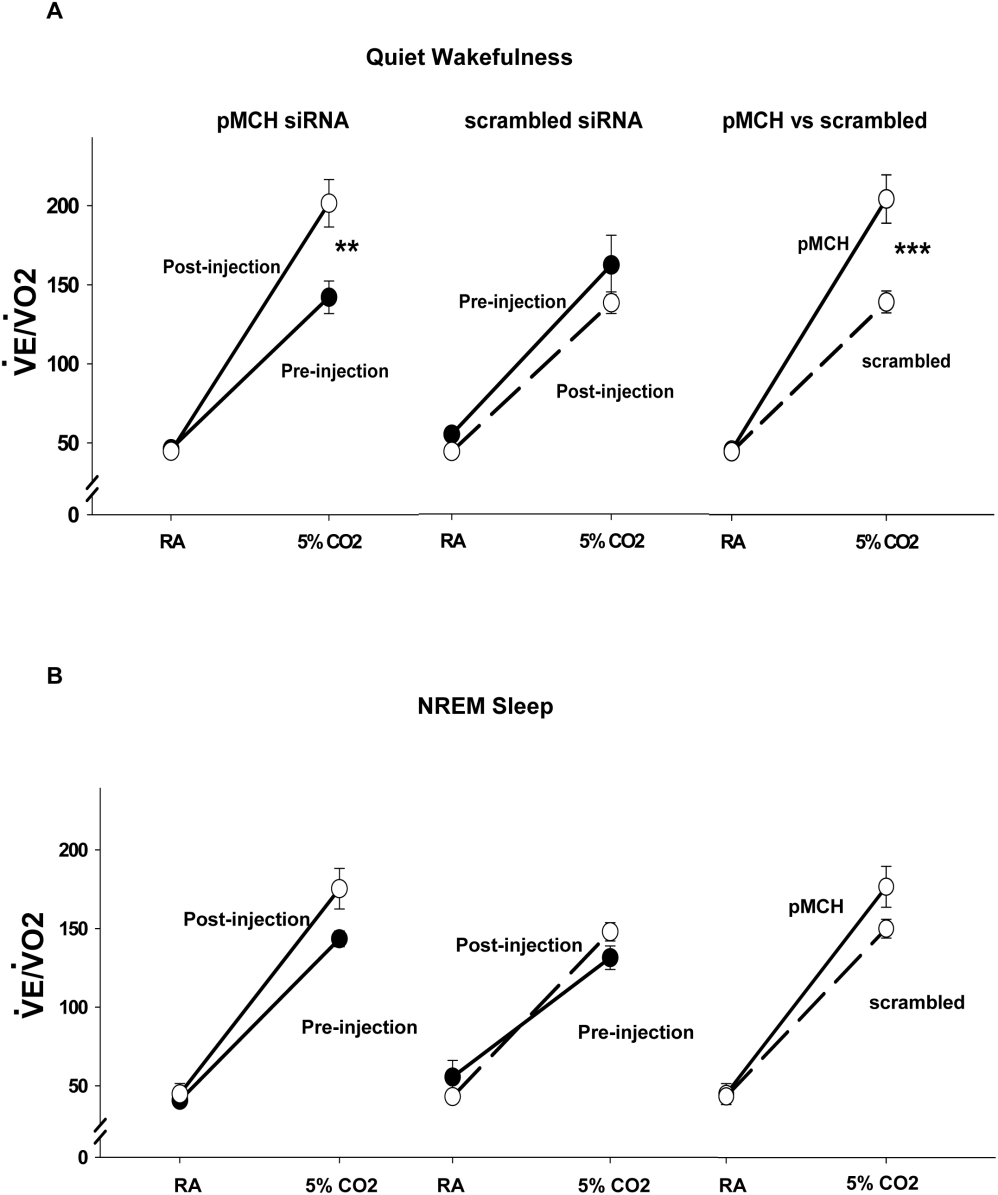
The effects of knockdown of pMCH mRNA expression on the 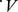
_E_/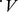
_O2_ response to hypercapnia. 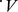
_E_/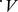
_O2_ ratio (Mean ± S.E.M.) in room air (RA) and 5% CO_2_ before (filled circles) and 72 hrs after (open circles) injections of pMCH siRNA or scrambled siRNA during quiet wakefulness (A) and NREM sleep (B) (N = 6; two way RM ANOVA, Bonferroni’s test). Significant difference is indicated with **(P<0.01) or ***(P<0.001).

Compared to the pre-injection baseline, pMCH siRNA induced a 42% increase in 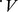
_E_/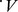
_O2_ while breathing 5% CO_2_ during quiet wakefulness (Fig. 3, left): before injection, 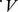
_E_/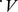
_O2_ breathing 5% CO_2_ was 142±10 ml min^−1^ (100 g)^−1^ and after injection was 202±15 ml min^−1^ (100 g)^−1^ (N = 6, P = 0.002, two way RM ANOVA, Bonferroni’s post hoc test). In the scrambled siRNA group, no significant difference was observed before and after scrambled-siRNA injection (Fig. 3A middle). There was no significant difference of 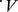
_E_/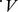
_O2_ in RA before and after siRNA injection in either group. Compared to the rats injected with scrambled siRNA, pMCH siRNA injection also induced a 47% higher 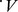
_E_/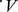
_O2_ in 5% CO_2_ (Fig. 3, right; N = 6, P<0.001, two way ANOVA, Bonferroni’s post hoc test): 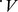
_E_/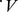
_O2_ was 202±15 ml min^−1^ (100 g)^−1^ and 137±7 ml min^−1^ (100 g)^−1^ in pMCH siRNA and scrambled siRNA groups, respectively. There were no significant changes in 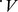
_E_/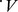
_O2_ induced by siRNA injections during NREM sleep in either RA or 5% CO_2_ (Fig. 3B). We also conducted a three way ANOVA with Bonferroni’s test to assess the dependence of the effects of treatment on gas components (RA or CO_2_) and vigilance states (wakefulness or NREM sleep). There is a significant interaction between treatment and gas components. In RA, no significant difference was found in 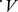
_E_/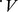
_O2_ comparing pMCH siRNA to scrambled siRNA treatment (P = 1); in 5% CO_2_, pMCH siRNA treated rats had a higher 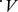
_E_/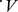
_O2_ than that in the scrambled siRNA group (P<0.001). The effects of pMCH siRNA were vigilance-state dependent; the effects on 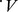
_E_/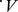
_O2_ were present only in wakefulness (P = 0.002); not in NREM sleep (P = 0.602).

**Figure 4:**
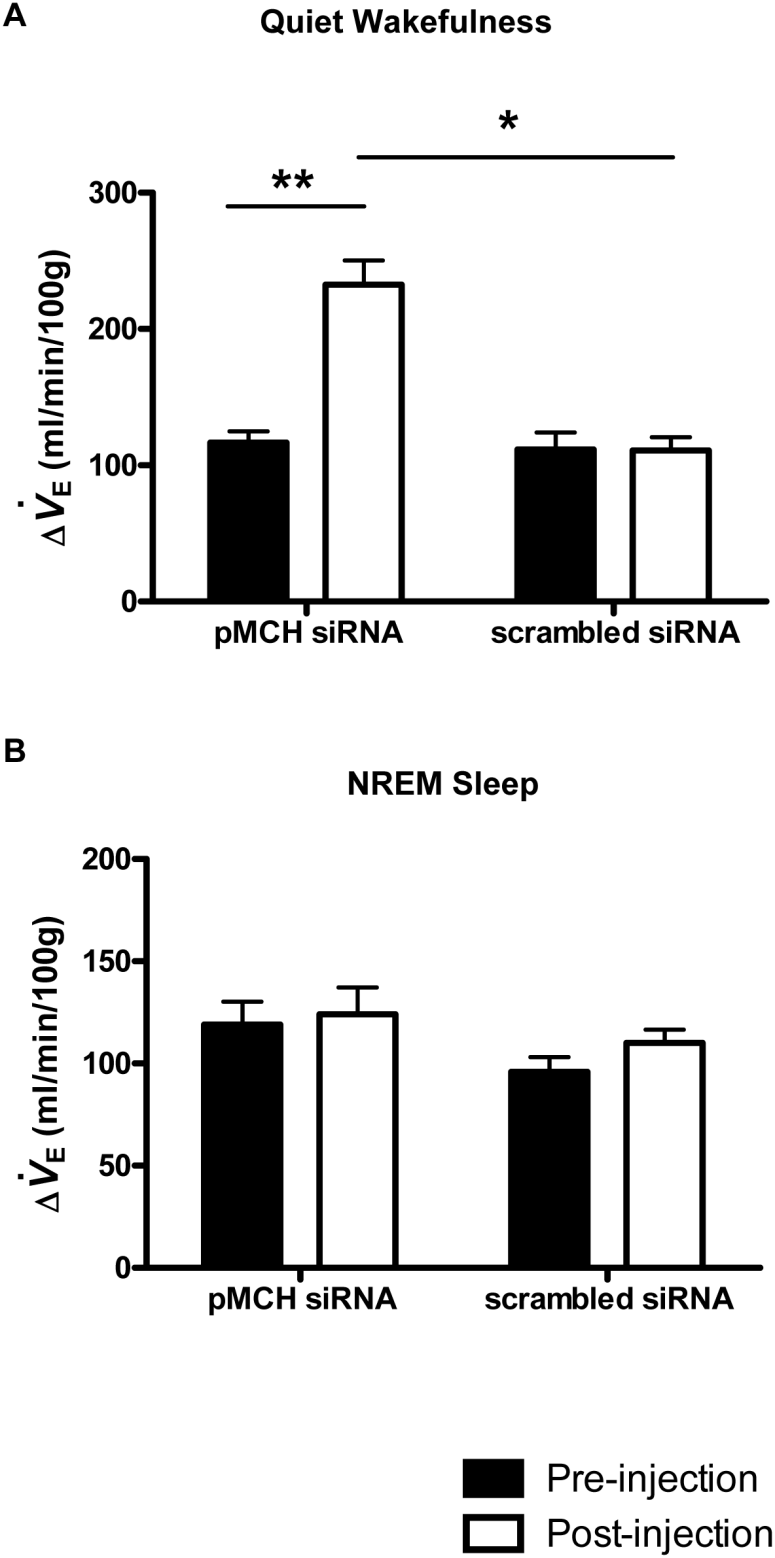
The effects of knockdown of pMCH mRNA expression on CO_2_ chemoreflex. Effects of treatments on the changes of ventilation from RA to CO_2_ (Δ
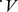
_E_: the difference between ventilation in RA and in 5% CO_2_). Δ
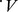
_E_ before (Filled bars) and 72 hrs after (open bars) pMCH or scrambled siRNA treatment during quiet wakefulness (A) and NREM sleep (B). (N = 6 each group, two way RM ANOVA, Bonferroni’s post hoc test) (Mean ± S.E.M.) Significant difference is indicated with *(P<0.05) or **(P<0.01).

Since 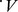
_O2_ was not affected by pMCH-siRNA the enhanced 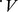
_E_/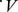
_O2_ response to hypercapnia induced by knockdown of pMCH was primarily due to increased ventilation. Fig. 4A shows that the CO_2_ response, shown as Δ
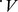
_E_, was enhanced after knocking down pMCH mRNA expression, from 117±8 ml min^−1^ (100 g)^−1^ pre-injection to 233±18 ml min^−1^ (100 g)^−1^ post-injection in quiet wakefulness (N = 6, P<0.001, two way RM ANOVA, Bonferroni’s post hoc test) but not in NREM sleep (Fig. 4B, N = 6, P = 1, two way RM ANOVA, Bonferroni’s post hoc test). No such effect was found in the scrambled siRNA injected group. The CO_2_ response in the pMCH group is significantly higher than that in the scrambled siRNA treated group (N = 6, P<0.001, two way ANOVA, Bonferroni’s post hoc test). The treatment effects on Δ
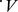
_E_ depended on what level of the vigilance state was present. There was a statistically significant interaction between treatment and vigilance state (P = 0.014). The significant effects were in wakefulness.

### Energy Balance

**Figure 5:**
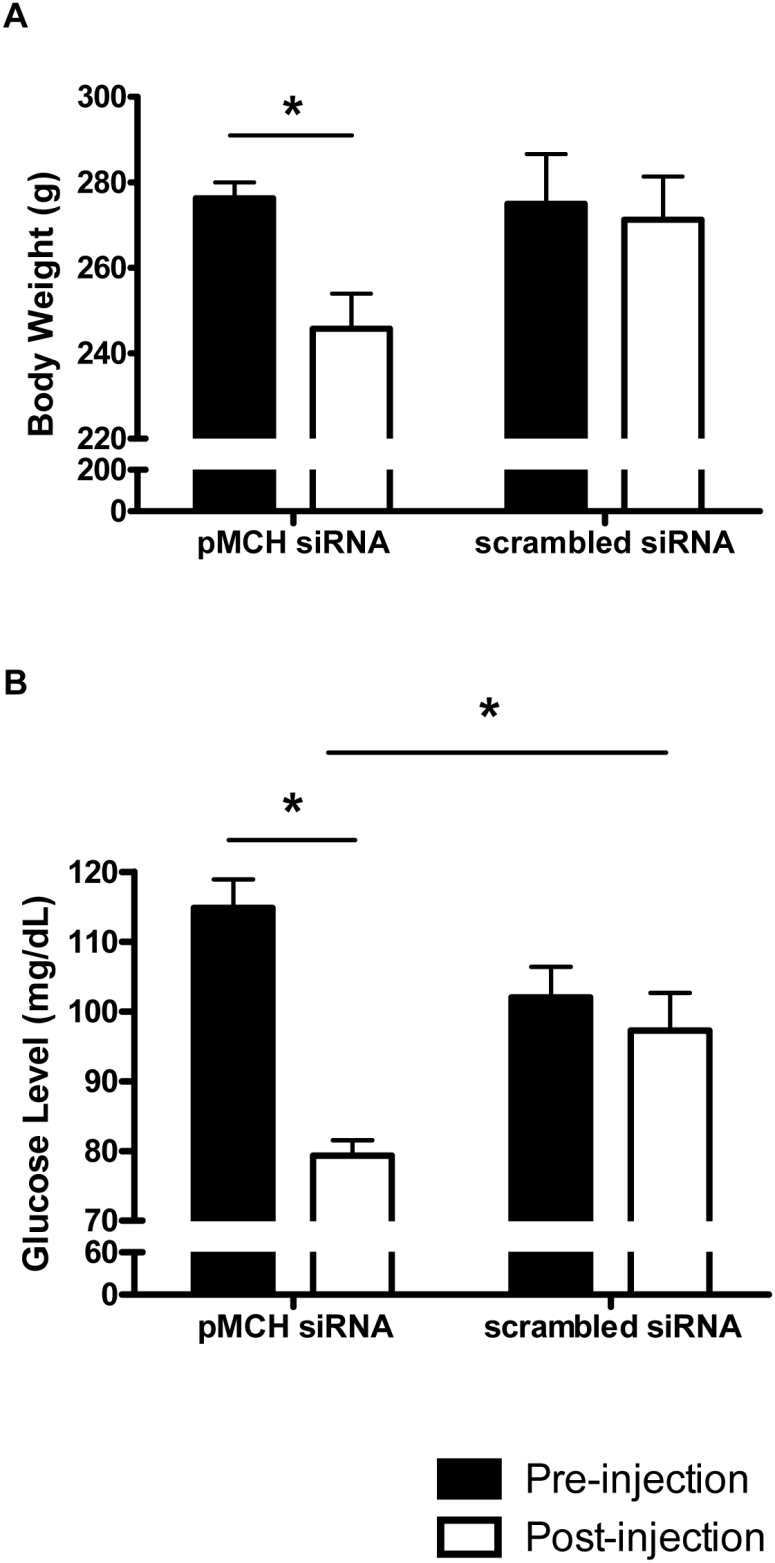
The effect of knockdown of pMCH mRNA expression on body weight and glucose levels. Body weight before (filled bars) and 72 hrs after (open bars) pMCH siRNA injection or scrambled siRNA injection (N = 6 each group, paired t-test) (A). Basal glucose levels before (filled bars) and 72 hrs after (open bars) pMCH siRNA or scrambled siRNA injection (N = 6 each group, paired t-test) (B). The pMCH siRNA treated group had significant lower glucose levels than that of scrambled siRNA treated group (B; one way ANONA). (Mean ± S.E.M.) Significant difference is indicated with *(P<0.05).

Fig. 5A shows that 72 hrs after bilateral injections of pMCH siRNA, the rats had experienced an ∼8% body weight loss; weight pre-injection, 275.5±6.5 g; weight post-injection, 252.9±7.7 g (N = 6, P = 0.046, paired t-test). In contrast, bilateral injection of scrambled-siRNA did not cause any significant weight changes. Fig. 5B shows that non-fasting basal glucose levels were reduced by pMCH-siRNA injection compared to pre-injection baseline. Glucose levels before pMCH siRNA injection were 115±4 mg/dl whereas after pMCH siRNA injection they dropped to 79±2 mg/dl, a 31% of decrease (N = 6, P = 0.031, paired t-test). This response was not observed in the scrambled siRNA treated group. There was no significant difference in the baseline glucose levels between pMCH siRNA and scrambled siRNA treated groups before injections (N = 6, P = 0.254, one way ANOVA, Bonferroni's post hoc test). However, after siRNA injection, pMCH siRNA treated rats had lower glucose levels compared to that in those receiving scrambled siRNA (N = 6, P = 0.012, one way ANOVA, Bonferroni's post hoc test).

Metabolic rate and body temperature were not affected by either treatment (data not shown).

## Discussion

In the present study, we demonstrated that specific knockdown of MCH in the CNS via injection of pMCH-siRNA in LHA led to: 1) an increase in the ventilatory hypercapnic chemoreflex in wakefulness but not NREM sleep; and 2) a decrease in body weight and basal blood glucose levels in conscious, freely-moving, and non-fasting adult rats.

### Knockdown pMCH with siRNA

RNAi is a widely used potent method to suppress specific gene expression in mammalian cells and study gene function. However a critical issue in siRNA design is to guarantee that the designed siRNA sequences are specific and free of off-target effect. The actual mechanism of off-target effect is still unclear, however it has been shown that a partial sequence homology between siRNA and its unintended target is one of the contributing factors [32], [33]. How to evaluate and mitigate the off-target effect is a very challenging issue particularly for the whole animal experiments and human clinical trials [32]. In this study, to verify the specificity and minimize the off-target effects we: 1) used a pool of three pMCH siRNAs to silence the pMCH gene; 2) measured both MCH mRNA expression and the protein level of MCH in the CNS in both knockdowns and controls; 3) measured, *in* *vivo*, the effects of MCH on respiratory response to hypercapnia and glucose in both control and knockdown animals; 4) used scrambled siRNA as one of the controls; 5) matched analysis of the pMCH siRNA sequences with the gene database at GenBank; 6) measured orexin-A mRNA expression, which is produced by the adjacent orexin neurons in the LHA. Pooling multiple siRNAs has been suggested as one way to reduce the off target effects due to competition among the siRNAs in the pool [32], [34]. Kittler *et al.,* reported that pooling of multiple siRNAs to the same target significantly reduced a number of cells and the magnitude of off-target silencing relative to a single siRNA *in* *vitro* [34]. Both *in* *vitro* MCH mRNA expression and MCH protein measurements, and *in* *vivo* functional measurements of the respiratory response to hypercapnia and changes of body weight and basal glucose level demonstrated that we have successfully knocked down pMCH in the LHA. Based on the match analysis of the pMCH siRNA sequences with the database at GenBank, the most likely off-target gene of the pMCH siRNAs is Poly [ADP-ribose] polymerase 1 (PARP1) binding protein. PARP1 is a product transcribed from the opposite strand of the MCH gene; however, PARP1 binding protein mRNA is not present in the rat hypothalamus [35]. The other possible off-target genes include TGFB-induced factor homeobox 2-like, X-linked 2 (Tgif2lx2), osteopetrosis associated transmembrane protein 1 (Ostm1), coiled-coil domain containing 124 (Ccdc124), UFM1-specific peptidase 2 (Ufsp2), and pericentrin (Pcnt), genes that are not physiologically relevant to MCH function. Therefore, we conclude that the observed change of ventilatory response to hypercapnia is due to knocking down of pMCH via pMCH siRNAs in the LHA.

Our approach of using siRNA to knockdown the expression of the pMCH mRNA was aimed at reducing the synthesis of MCH and producing a reduction in MCH levels in the CNS for a short period of time. Here we showed that the pMCH-siRNAs efficiently lowered pMCH mRNA expression by ∼62% in the LHA (Fig. 2A) and decreased MCH levels by ∼43% in the CSF (Fig. 2B) while the levels of mRNA expression of orexin-A and OX1R were not significantly affected by pMCH siRNA injections in the LHA (Fig. 2A). However, aside from MCH, two other peptides, neuropeptide-glutamic acid-isoleucine (NEI) and neuropeptide-glycine-glutamic acid (NGE), are also derived from the same common precursor pMCH [36], and knocking down the pMCH in the LHA may also reduce the levels of NEI and NGE in the CNS [2], [14], [36]. At the present time, limited studies on NEI and NGE have not demonstrated any biological and physiological significance [2], however, a recent report suggest that NEI may affect grooming behavior and motor activity [37].

### MCH and hypercapnic chemoreflex

Our present study has demonstrated for the first time in conscious animals that MCH is involved in central chemoreflex. Reduced expression of the pMCH mRNA, which resulted in a lower MCH level in the brain, led to an enhanced hypercapnic ventilatory chemoreflex as demonstrated by increased 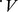
_E_/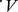
_O2_ and Δ
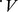
_E_ in rats in wakefulness (Fig. 3A & 4A). Since both metabolic rate and body temperature were not affected by pMCH siRNA, we conclude that the enhanced hypercapnic chemoreflex (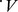
_E_/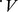
_O2_ and Δ
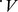
_E_) observed in this study is primarily due to the ventilatory change induced by knockdown of MCH expression. How MCH neurons participate in regulation of the central hypercapnic chemoreflex is not well understood at the present time. A study with injection of a rabies virus transneuronal tracer in the diaphragm revealed that MCH neurons have multisynaptic connections with the diaphragm motoneurons [11]. MCH projections and MCHR1 are found in many brain sites that contain central chemoreceptors, e.g., the RTN, NTS, LC, dorsal and medullary raphe nucleus [3], [12]–[15], [38]. Presumably MCH neurons can directly alter the neuronal activity in these putative chemoreceptor sites and thus change the chemosensitivity of the central chemoreflex, or directly or indirectly modulate the phrenic motor neuron activity.

In the LHA, MCH neurons are closely intermingled with chemosensitive orexin neurons [11], [16], [17], [19], [20], [39], [40], and have a reciprocal synaptic relationship with each other [41]. Orexins are predominantly excitatory neuropeptides [42], whereas MCH is primarily considered as an inhibitory neuropeptide [20], [43]–[45]. In brain slices, orexins directly and indirectly excite the MCH neurons [20] while MCH attenuates orexin-A induced excitation in orexin neurons [22]. Orexin and MCH neurons are physiologically antagonistic in many aspects. In respect to the ventilatory hypercapnic chemoreflex, inhibition of the orexin system decreased the CO_2_ chemoreflex [24], [31], while here we showed that decreased level of MCH in the CNS increased the ventilatory CO_2_ chemoreflex. We speculate that removing the inhibitory MCH influence on orexin neurons in the LHA by knockdown of pMCH further facilitates orexin excitatory effects on the central chemoreceptors and subsequently induces an increased central hypercapnic chemoreflex. That the enhanced hypercapnic chemoreflex was observed only in wakefulness (not in NREM sleep) during the experimental hour in the dark period in this study also indirectly support this hypothesis since orexin is most active in wakefulness during the dark period [46]. Injection of MCH into the spinal cord significantly decreased sympathetic responses to both anoxia and hypercapnia but the respiratory (phrenic activity) response remained unchanged in anesthetized rats (Egwuenu et al., 2012). In summary, our study suggests that MCH, in contrast to orexin, acts to suppress the central hypercapnic chemoreflex.

### MCH and body weight, glucose level, metabolic rate and body temperature

The rats treated with pMCH siRNA experienced an 11% body weight loss at ∼72 hrs after injection (Fig. 5A) in our study, however all rats were in good health with no signs of dehydration or stress. The weight loss resulted by decreased central MCH in this study is consistent with the general role of MCH on feeding and body weight as reported by other groups [4], [5], [47]–[51].

We also observed a significant reduction in the non-fasting basal glucose levels after knocking down pMCH mRNA (Fig. 5B). MCH is involved in the glucose regulation, which has been discussed in detail in many recent publications [5], [42], [52]–[54]. Overexpression of MCH in mice leads to obesity and insulin resistance [5], [54], and in contrast, pMCH^−/−^ and MCH-neuron-ablated mice are lean with improved insulin sensitivity [55], [56]. The decreased non-fasting basal glucose levels after knockdown of pMCH mRNA in our study may be the result of improved glucose metabolism induced by lower levels of central MCH.

### MCH and sleep homeostasis

MCH neurons discharge maximally during REM sleep and occasionally during NREM and are silent during wakefulness [46]. However, the mechanism by which MCH affects the sleep-wake cycle remains controversial at present time. We have decided not to draw any conclusions on the effect of knocking down MCH on the sleep-wake cycle for the following reasons. 1) Our current study was optimized to evaluate the maximal changes of ventilation in air-hypercapnic conditions in wakefulness and sleep at the beginning of the dark period, and therefore these data only reflected the changes of wakefulness-sleep during this particular period. 2) Our siRNA induced change of MCH in the CNS was short lasting. In our case, the lowest MCH mRNA expression was observed at 48 hr post injection, and it came back to normal after 72 hr. We think that it is very difficult to study the effect of MCH on sleep-wake cycle, which requires many hours of recording, while the level of MCH in the CNS is not stable; 3) To accurately address the issue of MCH knock-down effects on sleep will require a different experimental design with more than 24 hr continuous recording with steady level or levels of MCH in the CNS.

In conclusion, decreasing the level of MCH via knockdown of pMCH mRNA expression in LHA and in the CNS: 1) enhanced the ventilatory response to hypercapnia in wakefulness; and 2) induced weight loss and lowered basal non-fasting glucose levels in conscious rats. These data suggest that the neuropeptide MCH in the LHA potentially functions as a negative feedback modulator in regulation of the central chemoreflex and may be the link between energy homeostasis and autonomic function.
